# Cardiometabolic risk factors and neurodegeneration: a review of the mechanisms underlying diabetes, obesity and hypertension in Alzheimer’s disease

**DOI:** 10.1136/jnnp-2023-332661

**Published:** 2024-01-30

**Authors:** Vijay Patel, Paul Edison

**Affiliations:** 1 Department of Brain Sciences, Imperial College London, London, UK; 2 Cardiff University, Cardiff, UK

**Keywords:** Alzheimer’s disease, cardiometabolic risk factors, diabetes, hypertension, insulin resistance, neuroinflammation, obesity, reactive oxygen species

## Abstract

A growing body of evidence suggests that cardiometabolic risk factors play a significant role in Alzheimer’s disease (AD). Diabetes, obesity and hypertension are highly prevalent and can accelerate neurodegeneration and perpetuate the burden of AD. Insulin resistance and enzymes including insulin degrading enzymes are implicated in AD where breakdown of insulin is prioritised over amyloid-β. Leptin resistance and inflammation demonstrated by higher plasma and central nervous system levels of interleukin-6 (IL-6), IL-1β and tumour necrosis factor-α, are mechanisms connecting obesity and diabetes with AD. Leptin has been shown to ameliorate AD pathology and enhance long-term potentiation and hippocampal-dependent cognitive function. The renin-aldosterone angiotensin system, involved in hypertension, has been associated with AD pathology and neurotoxic reactive oxygen species, where angiotensin binds to specific angiotensin-1 receptors in the hippocampus and cerebral cortex. This review aims to consolidate the evidence behind putative processes stimulated by obesity, diabetes and hypertension, which leads to increased AD risk. We focus on how novel knowledge can be applied clinically to facilitate recognition of efficacious treatment strategies for AD.

## Introduction

Alzheimer’s disease (AD) is characterised by progressive neurodegeneration associated with synaptic dysfunction and neuronal death, which culminates in brain atrophy. Pathologically, this is characterised by the deposition of senile plaques composed of β-amyloid (Aβ) and neurofibrillary tangles (NFTs), formed of hyperphosphorylated τ. The accumulation and aggregation of abnormal proteins leads to microglia-mediated release of proinflammatory cytokines contributing to neuroinflammation, which is another key feature of neurodegeneration. These cytokines, such as interleukin-6 (IL-6) and tumour necrosis factor-alpha (TNF-α), lead to neuronal and synaptic dysfunction.^1^

Age is the most common risk factor for AD, which also has a strong genetic component. The apolipoprotein E ε4 (APOE ε4) allele is the greatest genetic risk factor for late-onset AD; the most common form of the disease. APOE ε4 exacerbates τ hyperphosphorylation and affects Aβ deposition and clearance.^2^

Cardiometabolic risk factors play a significant role in AD. Diabetes, obesity and hypertension are highly prevalent and can accelerate neurodegeneration and perpetuate the burden of AD. Projections show that by 2030, up to 20% of the world will be obese; while 85% of Americans would be overweight or obese. Over 450 million people were affected by diabetes worldwide in 2017 alone. The WHO states that 1.28 billion people live with hypertension worldwide. Qrisk2, a holistic score for cardiovascular disease risk, was associated with specific biomarkers of AD and clinical progression of the disease.^3^ APOE ε4 non-carrier participants with prodromal AD (early/late mild cognitive impairment (MCI)) and higher levels of cardiovascular risk exhibited greater Aβ deposition and pronounced glucose hypometabolism compared with low cardiovascular risk individuals.^3^ This demonstrates the significance of cardiometabolic risk factors on AD progression independent of APOE ε4 status.

Genome-wide association studies have identified that certain pleiotropic loci exist which link AD to cardiometabolic traits. The allele associated with reduced brain ACE was related to increased risk of developing AD. AD and type 2 diabetes mellitus (T2DM) share 395 single nucleotide polymorphisms related to cellular immunity, neuronal plasticity and signalling.^4^

Mechanistic differences on how obesity, diabetes and hypertension exert their influence on the acceleration of neurodegeneration have been postulated. Insulin resistance (IR) is a distinct pathophysiological link between T2DM and AD. Comparatively, leptin resistance is a key mechanism underlying obesity, which may increase the risk of AD. Peripheral inflammation, concomitant with obesity and diabetes, is directly related to AD, demonstrated by higher plasma and central nervous system (CNS) levels of IL-6, IL-1β and TNF-α in patients with AD.^1^ Animal models have demonstrated that systemic inflammation may lead to central inflammation due to microglial activation. Peripheral IL-6 is associated with inferior temporal cortical thinning. Peripheral cytokines may cross the blood brain barrier (BBB) to activate microglia, which in turn disrupts grey matter architecture leading to cognitive dysfunction. Peripheral inflammation may differentially accelerate progression of AD in obesity and diabetes given their phenotypic differences. Hypertensive pathology and its contribution to AD, has been investigated although less thoroughly. Cerebral atherosclerosis, central angiotensin-II neurodegenerative effects and upregulation of advanced glycation end-product receptor (AGE) may represent putative mechanisms.

This review aims to consolidate the evidence behind putative processes stimulated by obesity, diabetes and hypertension, which leads to increased AD risk. We focus on how novel knowledge can be applied clinically to facilitate recognition of efficacious treatment strategies for AD.

## T2DM and Alzheimer’s disease

The most common subtype, T2DM, is associated with hyperglycaemia and IR, owing to decreased insulin receptor sensitivity. The Rotterdam study showed a twofold increase in the risk of developing dementia for diabetics compared with non-diabetics.^5^

Metabolic dysregulation in the form of IR is a feature of AD. Insulin modulates neuronal activity via promotion of synaptic plasticity owing to its direct effects on long-term potentiation and depression (LTP and LTD, respectively) at hippocampal synapses. Exogenous insulin led to improved memory performance test scores. The role of insulin as an antioxidant, via activation of the nuclear factor erythroid 2-related factor 2 (Nrf2) promoter, is pivotal in preserving cognitive function.^6^ Insulin signals through its tyrosine kinase receptor, which phosphorylates enabling the recruitment of insulin receptor substrate 1 (IRS1) and 2 (IRS2). As a result, a sequence of signalling pathways ensues, including the activation of phosphoinositide 3-kinase (PI3K) and Akt, a serine/threonine protein kinase. Insulin signalling in glial cells is involved in the modulation of proinflammatory cytokine secretion and central response to glucose availability via Akt/protein kinase B (PKB) cell signalling.^7^ Akt itself is an essential component of insulin signalling given its regulation of mitochondrial function, glucose transport via glucose transporters (GLUT) and synaptic plasticity—while also phosphorylating glycogen synthase kinase-3β (GSK3β). GSK3β is involved in induction of glycogen synthesis and the pathological formation of NFTs and is inactivated on phosphorylation.^8^

### IR in AD

Impaired insulin signalling is a feature of AD. Initial work using Tg2576 mouse models showed diet-induced IR promoted AD pathology. Subsequent post-mortem brain tissue analysis studies have shown that patients with AD have deficiencies in insulin and insulin-like growth factor signalling. Reduced levels of IRS and Akt are observed, as well as a potentiation in the activation of GSK3β. This kinase phosphorylates τ at serine residues at multiple different sites, which have been demonstrated in vivo as one of these phosphorylation-viable sites.^9^

Under physiological conditions, insulin promotes Aβ clearance and thereby prevents its extracellular accumulation. Aβ oligomers induce central IR in human hippocampal neuronal cells via activation of TNF-α and inhibition of IRS. Aβ oligomers activate the c-Jun N-terminal kinases (c-JNK) pathway, which is responsible for the phosphorylation and subsequent degradation of IRS. Aβ activates GSK3β in APP-V7171x Tau-P301L bigenic mice (with combined Aβ and τ pathology), which accelerates τ pathology because of tyrosine phosphorylation of GSK3β itself— thereby implicating GSK3β in the Aβ cascade hypothesis of AD pathology.^10^

**Figure 1:**
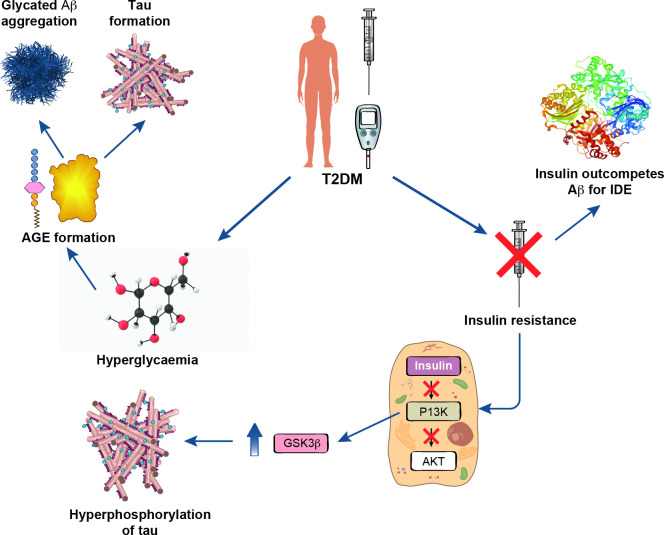
Schematic showing how type 2 diabetes mellitus influences Alzheimer’s disease via multiple mechanisms including insulin resistance and AGE formation. Aβ, β-amyloid; AGE, advanced glycation end products; Akt, a serine/threonine kinase; GSK3β, glycogen synthase kinase-3 beta; IDE, insulin degrading enzyme; T2DM, type 2 diabetes mellitus.

IR can affect tauopathy directly through GSK3β hyperactivity and indirectly through impaired Aβ extracellular clearance (figure 1). IR is a significant mechanistic link between T2DM and AD. Co-testing GSK3β gene expression profile and τ quantification in a future T2DM cohort would provide experimental validation of the putative mechanism. GSK3β inhibitors have shown promising results in mouse models demonstrating the therapeutic potential of Tideglusib (NP12) by reducing Aβ deposition, lowering the rate of τ phosphorylation and subsequently improving neuronal survival.^11^ A randomised clinical trial would be required to establish if this therapeutic targeting demonstrates adequate efficacy and safety, specifically in a diabetic cohort.

### Insulin degrading enzyme

Insulin degrading enzyme (IDE) plays a crucial role in regulating clearance of insulin and Aβ (figure 1). As well as being produced from direct proteolysis of amyloid precursor protein (APP), Aβ is also found intracellularly in the cytosol because of endosomal production. IDE is β-structure specific that prevents the formation of toxic oligomers. Its overproduction in genetically manipulated mice prevented Aβ pathology. Reduced proteolytic capacity exists in early stages of AD. Both insulin and Aβ are substrates for the active site of IDE. In diabetes, where a hyperglycaemic and hyper-insulinaemic environment exists, Aβ is outcompeted and remains toxic extracellularly.

Alternatively, systemically high levels of insulin may reduce insulin transport across the BBB with the effect of IR on IDE remaining unclear. Hyperglycaemia can cause an increase in nitrogen species, which in turn, leads to the pathological nitrogenation of IDE to form S-nitrosylated IDE, decreasing the enzymatic activity.^12^ Evidence from murine models and humans show significantly higher levels of S-nitrosylated IDE in patients with AD compared with controls.^12^ Nitrosative stress, a widely recognised feature of AD which follows a similar pathogenesis to oxidative stress (mitochondrial generation of nitrogen-containing-free radicals) has been shown to have no effect on the ability of IDE to bind insulin. This, added with the greater levels of circulating insulin in T2DM, could synergise to result in a collation of toxic Aβ which is less rapidly broken down.

IDE conformation to create an allosteric Aβ-specific targeting enzyme has been highlighted as a real opportunity. Existing work has shown how certain genetic mutations (eg, cysteine-free IDE mutant) can cause a downstream change in IDE active site morphology, which leads to a modulated degradative effect and altered Aβ aggregation propensities.^13^

### Advanced glycation end products

Hyperglycaemia is a cardinal feature of T2DM and plays a predominant role in certain disease complications due to its effect on blood vessels and nerves (figure 1). Hyperglycaemia is implicated in T2DM, and AD. High glucose conditions inhibit the degradation of APP allowing for more binding sites for the β-secretase enzymes to bind and cleave Aβ. AGEs are the outcome of a glycation reaction between sugars and proteins. In a hyperglycaemic environment, levels of AGEs increase dramatically. AGEs cause neurotoxicity and influence AD pathology due to their regulation of Aβ aggregation^14^ and τ phosphorylation.^15^ Immunohistochemically, AGE presence has been shown not only in senile Aβ plaques but also NFTs. APP and Aβ are upregulated via the effect of reactive oxygen species (ROS) because of AGE. It is not certain whether AGE-modified Aβ is a causative event or a secondary outcome of Aβ deposition.

### Mitochondrial dysfunction

A specific dysfunction to liver and muscle mitochondria in diabetic rodents is observed.^16^ The dysfunction exists as either bioenergetic or biogenesis defects or in morphology and number. Levels of peroxisome proliferator-activated receptor gamma coactivator 1-alpha (PGC-1α_, which regulates the expression of genes associated with mitochondrial replication are decreased in insulin-resistant patients. When PGC-1α is overexpressed in mice, glucose uptake and glucose transport proteins in skeletal muscle are increased. Subjects with type 2 diabetes have a smaller size and density of mitochondria compared with lean controls; specifically of subsarcolemmal mitochondria responsible for membrane and transport processes. This leads to decreased electron transport activity and signal transduction accentuating IR. Hippocampal mitochondria in diabetic rats show dysfunctional features including altered ATP production, fewer cristae and lower general numbers; all of which contribute to cognitive decline. Compensatory mechanisms such as mitophagy, which revolves around engulfing and ultimately breaking down certain deficient mitochondria, is less rife in cells exposed to hyperglycaemia conditions.^17^

Individuals with both AD and T2DM have oxidised damaged DNA, as well as oxidised protein and lipid products. It is debated whether oxidative stress induces neuroinflammation or rather accentuates an ongoing pathology. Previous work has supported the idea that T2DM linked mitochondrial dysfunction underlies the initiation of AD. Both high fat diets (HFD) T2DM-induced mice and 3xTg-AD mice exhibit similar patterns of mitochondrial dysfunction with similar rates of ROS production.^18^

Mechanisms underpinning the link between diabetic mitochondrial dysfunction and AD are based on decreased ATP production, dysfunctional or reduced mitochondrial cytochrome c oxidase activity and hyperglycaemia-induced ROS production. Mitochondrial ROS production increases Aβ production, which can be inhibited by administration of antioxidants.^18^ Aβ accumulation in the mitochondria causes mitochondrial swelling, increased mitochondrial membrane permeability and ROS overproduction. Mitochondrial dysfunction leading to an energy metabolism deficit, alongside the concomitant increased ROS production and reduced mitochondrial cytochrome c oxidase activity, underlie cognitive decline in AD. Though IR predisposes diabetics to AD, mitochondrial dysfunction may not be associated with IR-related changes. The levels of oxidative stress precede the cascade of events prior to the presence of brain IR.^19^ Dysfunctional mitochondria may cause oxidation resulting in accumulation of certain lipids and lipid mediators such as diacylglycerols (DAG) and ceramides (CER).^20^ DAG and CER inhibit insulin signalling pathway via inhibition of the protein kinase Akt. Targeting ROS and hydrogen peroxide through genetic manipulation, preserves glucose levels and insulin sensitivity, suggesting an interaction between mitochondrial dysfunction and IR.

### Peripheral inflammation in diabetes

Systemic low-grade inflammation is a common feature of T2DM. Markers of peripheral inflammation are positively associated with risk of T2DM. Peripheral inflammation is potentially linked to the induction of IR, which leads to AD sequelae. TNF-α is commonly increased in T2DM and is likely a key pathogenic influence connecting diabetics to an increased AD risk.^21^ TNF-α reduces expression of GLUT-4 which is regulated by insulin. Cytokine activation causes serine phosphorylation of IRS-1 leading to inhibition of insulin receptors, which in turn dampens the PI3K signalling pathway.

Peripheral inflammation has a direct effect on extracellular Aβ levels. Mouse models have shown that inflammation activates microglia, which results in reduced Aβ clearance. Peripheral inflammation influences induction of cerebrovascular dysfunction in a group of human-APOE ε4 expressing (ε4 FAD+) mice.^21^ BBB dysfunction leads to higher permeability that results in peripheral factors crossing into the brain such as TNF-α, IL-6 and IL-1.^22^ TNF-α affects Aβ deposition due to increases in APP, β-site APP-cleaving enzyme (BACE1) and γ-secretase expression. Peripheral IL-1 levels disrupt BBB function allowing crossing of further neurotoxic chemicals to change the microenvironment of neurons while impairing Aβ clearance.

AD and T2DM are characterised as protein-misfolding disorders—caused by misfolded Aβ aggregation, which promotes the misfolding of more proteins. In vitro and in vivo studies show that islet amyloid polypeptide (IAPP; also known as amylin)^23^ can potentiate Aβ aggregation by colocalising in brain parenchyma.^24^ The increased peripheral inflammation in diabetics causes greater BBB permeability, which allows peripheral IAPP to enter the brain to further propagate the Aβ aggregation.

Regarding therapeutic viability, the ELAD trial is evaluating the effect of liraglutide, an incretin mimetic, in participants with early AD.^25^ This is based on preclinical data suggesting that the glucagon-like peptide-1 (GLP-1) analogue reduces Aβ oligomers and normalises synaptic plasticity. GLP-1 analogue improves cognitive function, decreases Aβ oligomer levels and inflammation and enhances LTP. Liraglutide improved spatial memory, synapse number and reduced inflammation and plaque load. Two current studies EVOKE (https://classic.clinicaltrials.gov/ct2/show/NCT04777409) and ISAP (https://www.isrctn.com/ISRCTN71283871), are randomised double-blind placebo-controlled clinical trials evaluating safety of semaglutide and its efficacy of reducing cerebral τ levels and inflammatory levels.^26^

### Obesity and AD

Obesity is defined as body mass index (BMI)>30 kg/m^2^ and is associated with an energy imbalance caused by high caloric intake and/or low energy expenditure. A recent study from the English Longitudinal Study of Ageing indicated a 31% greater risk of developing dementia in obese individuals from a cohort of 6582 subjects.^27^ Similar to diabetes, the exact mechanistic link remains unclear. Obesity is linked to systemic IR caused by hyper-release of insulin from β cells of the pancreas. Adipose tissue, being an active endocrine organ, releases proinflammatory adipokines and cytokines that become exaggerated in obesity creating a systemic low-grade inflammatory environment. These include leptin, adiponectin, TNF-α and IL-6. APP has recently shown to be upregulated within adipocytes in obesity and its levels correlate with adipocyte cytokine expression levels.^28^ Fat-fed mice show a phenotypic switch from anti-inflammatory M2 to proinflammatory M1-macrophages, and these cells then secrete TNF-α and IL-6. The proinflammatory state may lead to BBB dysfunction, triggering a central inflammatory response. Another possible mechanism linking obesity to AD is leptin resistance, a hallmark feature of obesity. The hormone has neuroprotective properties in preserving memory with its influence on hippocampal functioning. It is plausible that hyperleptinaemia may contribute to cognitive deficits. This hypothesis awaits experimental validation.

### Peripheral inflammation in obesity

**Figure 2:**
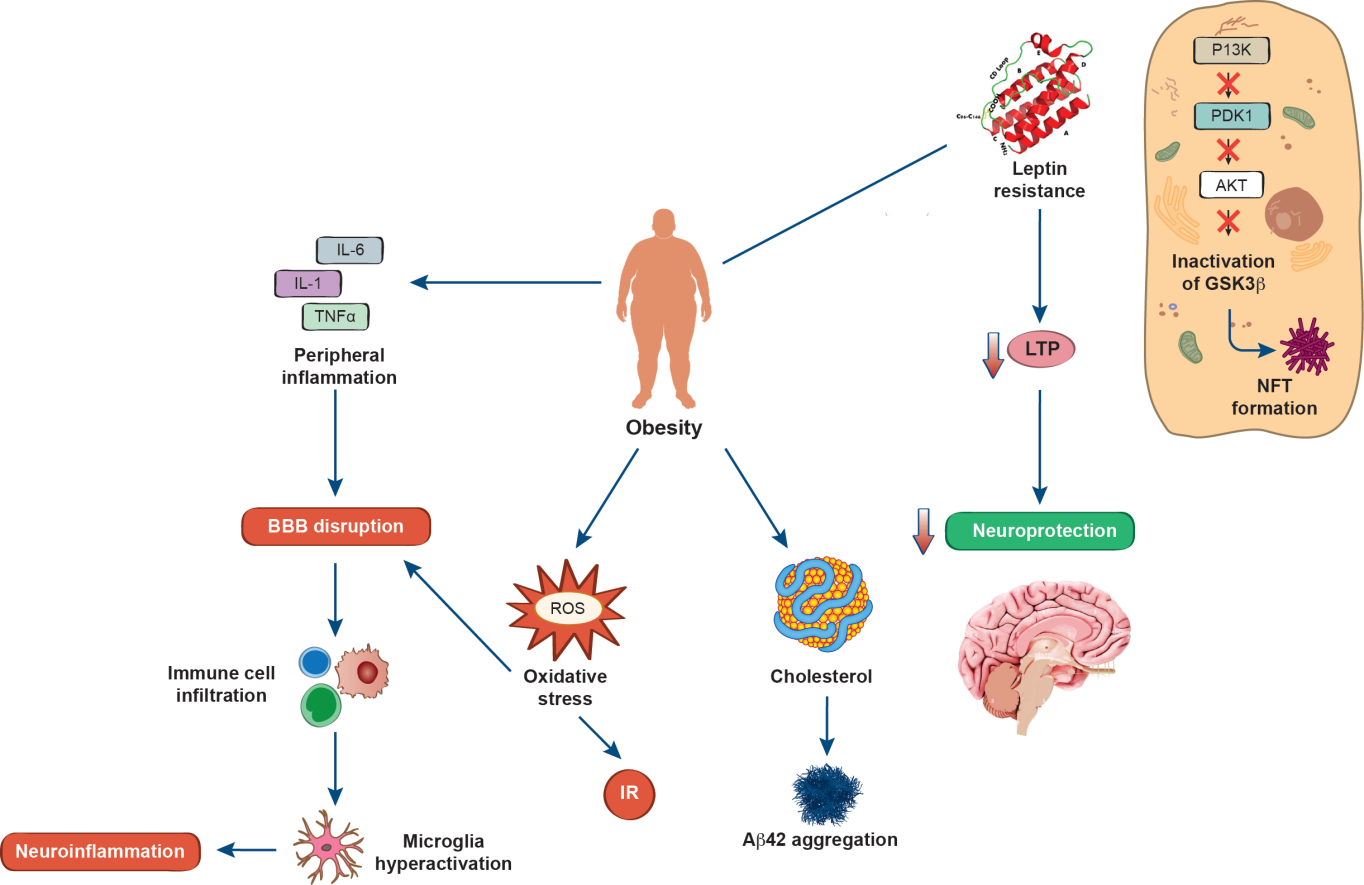
Schematic showing downstream effects of obesity on neurodegeneration via multiple mechanisms including oxidative stress, BBB disruption, leptin resistance and inflammation. Aβ, β-amyloid; Akt; a serine/threonine kinase; BBB, blood brain barrier; GSK3β, glycogen synthase kinase-3 beta; IL-1/6, interleukin-1/6; IR, insulin resistance, LTP, long-term potentiation, NFT, neurofibrillary tangles; PDK1; phosphoinositide-dependent kinase-1; PI3K, phosphoinositide 3-kinase; ROS, reactive oxygen species; TNF, tumour necrosis factor.

Obesity, like diabetes, is associated with peripheral inflammation via multiple cytokine releases including IL-1, IL-6 and TNF-α^28^ (figure 2). HFD, typical to these conditions, cause neuroinflammatory changes, oxidative stress and cognitive impairment. Neuroinflammation owing to persistent and aberrant microglial activation is a well-established pathogenesis concerning AD. Using APP^NL-G-F^ mice, peripheral inflammation increases BBB permeability to increase immune cell infiltration, which leads to microglial hyperactivation inducing neuroinflammation.^29^ A link between peripheral inflammation and levels of Aβ deposition in the mouse brain has been demonstrated, which may be caused due to microglial dystrophy impairing Aβ clearance.

Higher BMI levels are correlated to higher levels of circulating IL-6 in obese patients.^28^ IL-6 has an inverse relationship with hippocampus (HC) grey matter volume. This may be caused by an inflammatory stimulus resulting in HC glial cells producing IL-6. This subsequently inhibits neurogenesis and disrupts synaptic plasticity. Tocilizumab-treated (anti-IL-6R) cognitively impaired mice showed cognitive improvement and reduction in Aβ immunoreactivity.^30^ Future studies should investigate the potential of anti-IL-6 therapies in obese patients with AD. Obesity is related to higher levels of circulating cortisol as systemic inflammation triggers an increase in blood cortisol. High cortisol levels are associated with atrophy of temporal lobe parts, which is a feature of AD.

### Oxidative stress in obesity and diabetes

Chronic consumption of high fat causes an excess build-up of energy substrates in cells leading to increased ROS production (figure 2). ROS promotes oxidative stress in certain cells by damaging DNA, cellular proteins and the cell membrane while possessing the ability to modulate Nuclear Factor Kappa B (NF-κB) activity inducing an inflammatory state. Both β cells and neuronal cells have reduced antioxidant capabilities and are prone to ROS-mediated damage. Oxidative stress increases susceptibility to IR that has detrimental neuronal implications, but directly impacts neuronal cell health. Mitochondrial targeted antioxidants (the mitochondria are a major site of ROS production) preserved insulin sensitivity in rats fed on an HFD.^31^

Ferroptosis is a type of cell programmed death which is iron dependent. One hallmark characteristic of ferroptosis is production of ROS.^32^ Ferroptosis is implicated in induction and progression of T2DM. Excess iron stores are associated with T2DM and higher ferritin levels. Excess intracellular iron negatively impacts gene expression in pancreatic β cells leading to their dysfunction. nuclear assenbly factor-1 (NAF-1), a regulator of iron levels, has a reduced expression in certain β cells which paints a ferroptosis-like picture.^33^ Ferrostatin-1 treatment reduces levels of ferroptosis in these cells.

Ferroptosis is a key feature of AD.^32^ Accumulation of lipid peroxides, which is another characteristic of ferroptosis, is seen in AD. They tend to localise with Aβ plaques and are associated with disease acceleration. Lipid ROS generation, as aforementioned, is another characteristic of ferroptosis and is also present in excessive toxic levels in AD conditions. Ferroptosis inhibitors have been investigated in clinical trials and showed promising results. An iron chelator, deferoxamine, decreased the rate of decline in a cohort of patients with AD.^34^ Perhaps ferroptosis inhibitors would prove disease modifying in a cohort of patients with diabetic AD by decreasing ROS production and preserving neuronal health.

Rodents fed on an HFD have offspring with BBB impairments, which is composed of endothelial, epithelial and tanycytic cells.^35^ In overweight or obese individuals, BBB is compromised as determined by increased cerebrospinal fluid (CSF) albumin compared with serum levels. HFD-fed rats specifically show changes to their tight junction proteins such as claudin-5 and claudin-12 with other mouse models showing obesity-induced downregulation of cytoskeletal proteins: vimentin and tubulin; both of which are involved in nutrient transport.^36^ Increased oxidative stress due to obesity contributes to perturbed BBB functioning. Oxidative stress, marked by levels of ROS, correlated to a biomarker of BBB disruption, S100β, in obese individuals undergoing high intensity workouts.^37^

### Cholesterol and Aβ

Since APOE ε4 is an important cholesterol regulator, cholesterol has been implicated in AD. Lipid membranes containing cholesterol contribute to Aβ42 aggregation by affecting primary nucleation because of multiple cholesterol interactions with the AD pathological peptide. Spontaneous Aβ42 aggregation is a slow process, but lipids such as gangliosides and sphingomyelin affect Aβ42 oligomerisation.^38^ Cholesterol increases affinity for APP and Aβ. Statin use is associated with reduced AD incidence, particularly Caucasians. In Hispanic and black cohorts only, lipophilic statins readily cross the BBB, which may disrupt the detrimental interaction between cholesterol and Aβ aggregation to ameliorate AD pathology.^39^ Clinical trials using simvastatin showed no symptomatic benefit for AD despite the statin lowering cholesterol. The discrepancies in findings between research propounds the ambiguity surrounding the translation of preclinical cholesterol-implicating AD studies to actual clinical relevance. Different statins or high dose statin administration is a potential avenue of future work.

### Leptin and AD

Leptin hormone levels correlate with the amount of body fat and are elevated in the CSF and hippocampus of patients with AD. Leptin receptor messenger RNA is downregulated in patients with AD, implicating leptin resistance in AD pathology.^40^ Plasma leptin levels have been associated with CSF Aβ and with the diagnosis of AD confirmed by CSF biomarkers. This suggests a molecular interplay between leptin metabolism and brain Aβ deposition.^41^ Leptin hormone induces hippocampal synaptogenesis as db/db mice that lack leptin receptors exhibit reduced spinal density of CA1 and CA3 neurons. LTP is a form of plasticity whereby there is a resultant persistent prolongation of synaptic transmission, where it is chronically reduced in LTD. Leptin has been linked to the enhancement of LTP and improving hippocampal-dependent cognitive function. Leptin inhibits Aβ-driven neuronal cell death via phosphorylation of transcription-3 (STAT-3). Endophilin-1, a synaptic protein upregulated in AD by Aβ-mediation, was countered by leptin treatment.^42^ Leptin protects neurons against ischaemic damage in mouse models, which demonstrates its neuroprotective ability (figure 2). Leptin treatment reversed an artificially induced β-amyloidogenic pathway through reduction of BACE-1 expression. Levels of phosphorylated τ decreased because of reduced activation of GSK3β. This study used rabbits where AD-pathology was induced by high cholesterol diets, which altered leptin signalling; thus, corroborating leptin involvement.^43^

Leptin replenishment therapy research in AD cohorts is a potential disease-modifying therapy. Caution should be exercised with such investigations given the depth of research linking leptin to oesophageal and breast cancer. There is paucity of research into the efficacy and safety of this pluripotent hormone in patients with MCI and AD.

### Hypertension and AD

Hypertension, defined by a persistently high systolic (>130 mm Hg) or diastolic (>80 mm Hg), affects an estimated 1.4 billion people worldwide. Despite the availability of low-cost and well distributed anti-hypertensive medications, there remains a large population with untreated hypertension, which perpetuates the burden of hypertension. Hypertension is the main modifiable risk factor for cardiovascular disease, including myocardial infarctions and heart failure. However, hypertension, being an arterial disease, is an identifiable risk factor for multiple other pathologies including renal dysfunction, retinopathy and cerebrovascular disease (stroke and vascular dementia). The pathophysiology linking hypertension to the aforenamed cerebrovascular diseases are well established; with hypertension promoting formation of atherosclerotic plaques and arterial smooth muscle hypertrophy in stroke and transient ischaemic attacks because of turbulent flow.^44^ In vascular dementia, hypertension-induced white matter (WM) lesions can result in cognitive deficits. Hypertensions linked to WM damage arises from a compromised BBB and occurrence of multiple small strokes (lacunar infarcts). Given the link between hypertension and vascular dementia, risk of cognitive decline is decreased because of anti-hypertensive treatment.^45^ These results may be translatable to AD. The presence of lacunar infarcts correlates with a greater likelihood of manifesting symptoms linked to AD.^46^

### Atherosclerosis involvement in hypertensive AD

Atherosclerosis, defined as the narrowing of arteries because of build-up of cholesterol plaques called atheromas, is closely associated with vascular dementia.^47^ Cerebral atherosclerosis and arteriolosclerosis are associated with AD and the low scores obtained in cognitive domains.^48^ Cerebral vessel pathology may be an under-recognised risk factor for developing AD. White matter hyperintensities (WMH) are brain lesions, usually identified by brain MRI, that indicate cerebral vessel disease and are closely linked to arterial hypertension.^49^ WMH have been associated with brain atrophy and reduced cognition specifically in AD.^50^ The Aβ-associated, white matter hyperintense regions strongly correlated with lobar cerebral microbleeds suggesting that cerebral amyloid angiopathy contributes to the relationship between Aβ and WMH.^51^ This supports a role for uncontrolled hypertension on AD manifestation. The branched and circular nature of the cerebral artery structure makes it prone to atherosclerosis. Hypertension promotes this cerebrovascular atherosclerosis which, in turn, causes cerebral hypoperfusion and oxygen deprivation. Circle of Willis atherosclerosis is a possible risk factor for AD due to its influence on levels on neuritic senile Aβ plaques and density of τ containing NFTs.^52^ Increases in Aβ plaque and NFT density occur because of circle of Willis atherosclerosis. The atherosclerosis causes a limited blood flow and a hindered clearance of neurotoxic molecules such as the AD-associated toxins. Brain hypoxia has been linked to increased APP cleavage culminating in increased resultant Aβ.^53^ Cardiac arrest patients have greater levels of Aβ because of ischaemia. Aβ may promote the formation of further atheromas in the circle of Willis creating a vicious cycle. APP cleavage to form Aβ induces nitric oxide synthase and macrophage recruitment (key cell in formation of atheroma); thus, demonstrating this sequence to be proinflammatory and atherosclerotic. Moreover, models using IDE deficient mice to decrease Aβ clearance resulted in these mice having larger pools of Aβ and large atherosclerotic lesions. It is therefore likely that Aβ and atherosclerosis work simultaneously to deleteriously effect cerebral perfusion which, as aforementioned, furthers Aβ cleavage. Further research is warranted into the in vivo specific mechanism that links increased Aβ to larger atheromas.

### Neuroinflammation crosslink between hypertension and AD

Neuroinflammation in AD revolves around the activation of glial cells, microglia and astrocytes, as a result of an inflammatory state mediated by the presence of Aβ plaques, NFTs and damage-associated molecular patterns.^1^ Microglia, being the resident macrophage of the CNS, drive the immune response to the inflammatory state while also being able to secrete neurotrophic factors and facilitate synaptic pruning. Activated microglia, release both neurotoxic molecules including ROS as well as immune signalling molecules (cytokines, chemokines, etc) which, in turn, can also activate astrocytes. The release of toxins from these glial cells induces neuronal cell death as well as stimulating further glial activation, in a vicious cycle. Hypertension is a potential risk factor for neuroinflammation. Hypertension-mediated vascular damage at the BBB may allow proinflammatory molecules such as TNF-α, IL-1β to enter the brain and activate glial cells thereby facilitating neuronal cell death. Chronic hypertension induced Tg-SwDI mice administered N-nitro-L-arginine showed a positive association between blood pressure with BBB leakage and cognitive deficits.^54^ This strengthens the role of neuroinflammation mediation in hypertensive patients with AD. Moreover, cerebrovascular microgliosis was increased at 6 months post induction of hypertension. The mice observed increased microvascular Aβ deposition. This highlights a potential interaction between hypertension and microvascular Aβ deposition. This may also be due to increased peripheral cytokine infiltration following BBB leakage. Hypertensive mice with transverse aortic coarctation show persistent microglia activation accompanied with significant Aβ deposition. Regarding therapeutic potential, an alleviation of Aβ burden is observed when administering lipopolysaccharide, an immune system activator, but not when inhibiting the immune system using ibuprofen. This indicates that inflammation, at an appropriate time, may be beneficial in halting vascular-associated AD pathology. Hypertension in mice induces cerebral hypoxic changes because of reduced cerebral blood flow. Subsequently, this led to greater activation of IL-1β, higher levels of detected hyperphosphorylated τ and neurodegeneration of the hippocampus, along with increased ROS.^55^ Hypertension-induced hypoxia leads to compensatory neuronal neuroglobin downregulation. This in turn leads to downstream generation of oxygen free radicals, which activates microglia-derived neuroinflammatory factors, including those with the ability to phosphorylate τ such as p38 mitogen activated protein kinase.

### Renin angiotensin system involvement in AD

**Figure 3:**
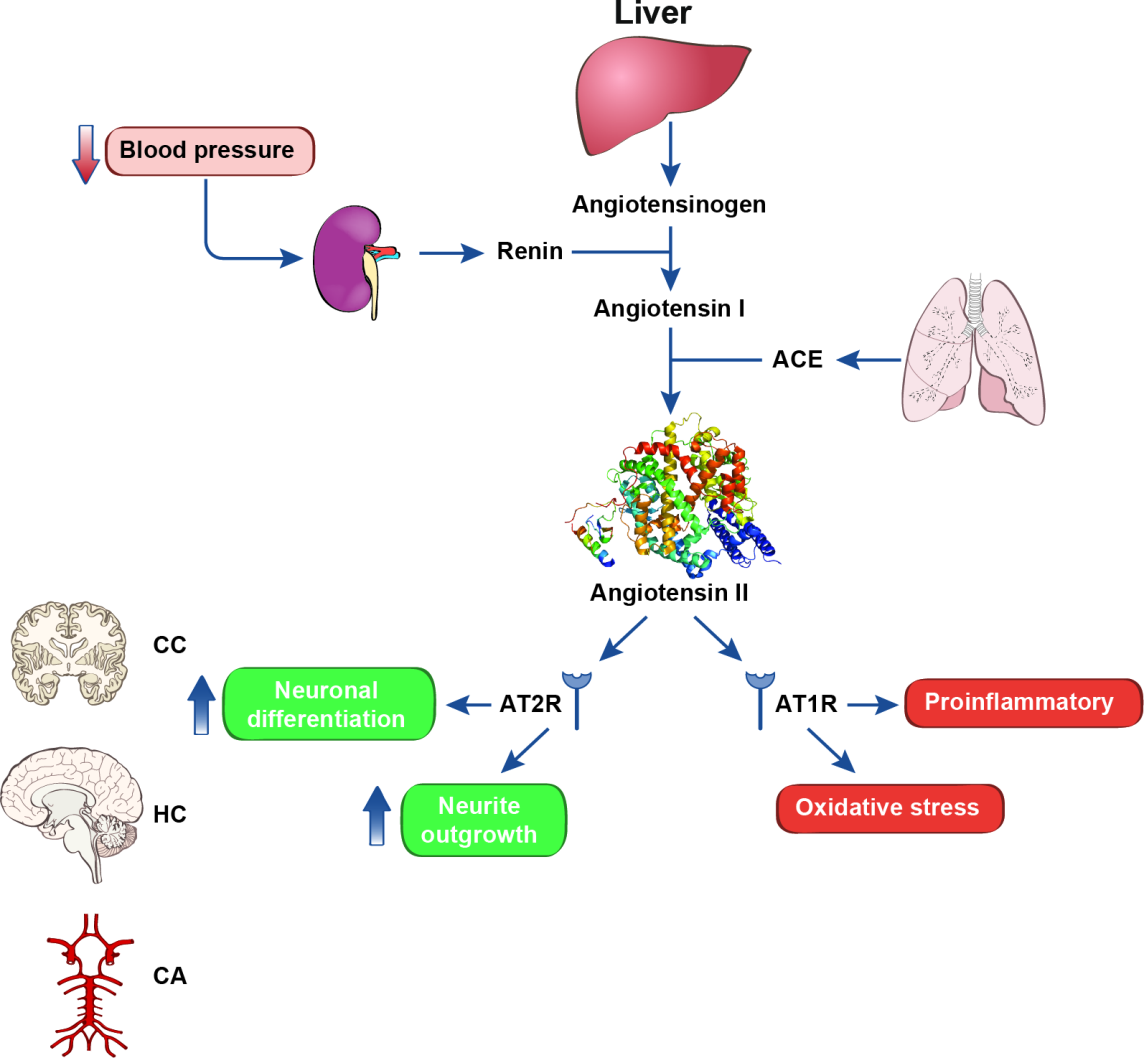
Schematic showing renin-angiotensin system can impact neurodegeneration via multiple mechanisms including oxidative stress and inflammation. AT1/2R, angiotensin-II 1/2 receptor; CA, cerebral arteries; CC, cerebral cortex; HC, hippocampus.

The endocrinological renin angiotensin system (RAS) ensures maintenance of homeostatic blood pressure through the release of renin because of a detection of low blood pressure at the juxtaglomerular cells of the afferent arterioles of the kidneys. Renin leads to the production and secretion of angiotensin-II (Ang-II), which increases blood pressure by promoting vasoconstriction and salt retention in the kidneys. Aberrant Ang-II production can lead to excess salt retention and, as a result, sustained hypertension. Evidence indicates the central involvement of Ang-II in AD, stemming from the interplay between the RAS and CNS glial cells. Ang-II binds angiotensin type 1 and type 2 receptors (AT1/2Rs) in cerebral cortex, hippocampi, basal ganglia and on cerebral blood vessels. AT1R is a proinflammatory pathway activator (figure 3). AT1R knockout mice have small size infarct size following middle cerebral artery occlusion. Activation of this receptor is associated with cognitive impairment. Ang-II administered to mice culminates in hippocampal gliosis. Greater levels of the peptide hormone are associated with lower total grey matter and hippocampal volumes. Ang-II serves as a proinflammatory mediator between hypertension and neuroinflammation.^12^ The therapeutic viability of using the antihypertensives angiotensin receptor blockers (ARBs) is likely fruitful given that preclinical studies in AD mouse models have shown the attenuation of cerebrovascular and neurological deficits due to ARB administration.^56^ Patients with mild-to-moderate AD treated with losartan (ARB) demonstrated no differences between cohorts in brain volume loss, white matter hyperintensities and cognitive assessments. Clinical trials investigating effectiveness of different ARBs over longer time periods and in cohorts of more severe AD may show promising data translated from preclinical studies.

AT1Rs are linked to hyperproduction of ROS through the effect on nicotinamide adenine dinucleotide phosphate (NADPH) oxidase activity. Oxidative stress is a key feature of AD and is regulated by the senile extracellular Aβ plaques.^56^ Losartan can improve cerebrovascular function in an Aβ-induced oxidative stress mouse model because of AT1R blockade providing an antioxidant effect. AT2R counteracts AT1R effects by regulating protein kinases and phosphatases that influence neuronal differentiation and regeneration.^19^ AT2R is upregulated during inflammatory responses in the brain as part of a compensatory mechanism. AT2R activation decreases TNF-α levels and increases the anti-inflammatory cytokine IL-10 in astrocytes and microglia and inhibits glial activation. In vitro and in vivo findings show that AT2R stimulation induce an inhibitory effect on NFkB and attenuate proinflammatory IL-6 levels. AT2R has opposite effects to those of AT1R on Aβ and τ pathology. AT2R reduces Aβ deposition and Aβ-induced neuronal damage.^19^ It is likely that AT2R may control the equilibrium between τ phosphorylation and dephosphorylation.

Ang-II is associated with aberrant hyperphosphorylation of tau.^56^ Central Ang-II significantly elevated the levels of p-tau in rodent brains, and this was accompanied with cognitive impairment. These effects were mediated by AT1R stimulation and subsequent activation of GSK3β. Therapeutic potential of losartan was highlighted due to its administration leading to attenuation of further τ phosphorylation and cognitive improvement.

## Conclusion

Cardiometabolic risk factors including diabetes, obesity and hypertension are associated with AD risk and incidence. Diabetes and obesity share common mechanisms modifying AD risk, such as IR-mediated GSK3β activation, AGE-mediated Aβ production and peripheral inflammation-mediated neuroinflammation. Leptin resistance is emerging as a key contributor to AD pathology with its effect on depressing LTP in hippocampal neurons. This leads to a lack of STAT3 phosphorylation causing more Aβ-driven apoptosis. Future work should endeavour to explore leptin replenishment as a viable therapy in an AD-targeted population. Hypertension involvement in AD is centred around the circle of Willis atherosclerosis, neuroinflammation and deleterious effects of Ang-II on τ phosphorylation and ROS generation. Losartan-based studies are warranted to determine if blocking certain AT1/2Rs would prevent p-tau and ROS build up.

Though certain promising preclinical drug-based therapies have failed in clinical studies, such as losartan, there is emerging preclinical data surrounding new potential therapies, including ferroptosis inhibitors and semaglutide, that warrant clinical investigation with the hope to discover a viable therapy for debilitating neurodegenerative diseases including AD.
